# Granulysin-Expressing CD4^+^ T Cells as Candidate Immune Marker for Tuberculosis during Childhood and Adolescence

**DOI:** 10.1371/journal.pone.0029367

**Published:** 2011-12-27

**Authors:** Henrik Mueller, Kellen C. Faé, Klaus Magdorf, Christian A. Ganoza, Ulrich Wahn, Ute Guhlich, Cornelia Feiterna-Sperling, Stefan H. E. Kaufmann

**Affiliations:** 1 Department of Immunology, Max Planck Institute for Infection Biology, Berlin, Germany; 2 Department of Pediatric Pneumology and Immunology, Charité University Medicine Berlin, Berlin, Germany; Fundació Institut d'Investigació en Ciències de la Salut Germans Trias i Pujol, Universitat Autònoma de Barcelona, CIBERES, Spain

## Abstract

**Background:**

Granulysin produced by cytolytic T cells directly contributes to immune defense against tuberculosis (TB). We investigated granulysin as a candidate immune marker for childhood and adolescent TB.

**Methods:**

Peripheral blood mononuclear cells (PBMC) from children and adolescents (1–17 years) with active TB, latent TB infection (LTBI), nontuberculous mycobacteria (NTM) infection and from uninfected controls were isolated and restimulated in a 7-day restimulation assay. Intracellular staining was then performed to analyze antigen-specific induction of activation markers and cytotoxic proteins, notably, granulysin in CD4^+^ CD45RO^+^ memory T cells.

**Results:**

CD4^+^ CD45RO^+^ T cells co-expressing granulysin with specificity for *Mycobacterium tuberculosis* (Mtb) were present in high frequency in TB-experienced children and adolescents. Proliferating memory T cells (CFSE_low_CD4^+^CD45RO^+^) were identified as main source of granulysin and these cells expressed both central and effector memory phenotype. PBMC from study participants after TB drug therapy revealed that granulysin-expressing CD4^+^ T cells are long-lived, and express several activation and cytotoxicity markers with a proportion of cells being interferon-gamma-positive. In addition, granulysin-expressing T cell lines showed cytolytic activity against Mtb-infected target cells.

**Conclusions:**

Our data suggest granulysin expression by CD4^+^ memory T cells as candidate immune marker for TB infection, notably, in childhood and adolescence.

## Introduction

Tuberculosis (TB) remains a leading cause of childhood mortality worldwide with 300,000 cases per year estimated globally and an unequal preponderance in developing countries [1], [2], [3]. While intense research efforts have focused on TB in adults, childhood TB has been largely neglected. Young children are at a much higher risk of progressing to active disease than adults. This is combined with higher mortality, with the highest prevalence among children under 2 years of age and the lowest between 5 and 10 years [4], [5]. Children also have a higher risk of extrapulmonary disease [6]. Due to its paucibacillary nature, diagnosis of childhood TB remains challenging. Most children with active TB are sputum smear negative. Less than 10–15% of children with proven TB exhibit sputum smear positivity for acid-fast bacteria [7]. The Mantoux tuberculin skin test (TST) in children also results in poor specificity, especially in countries where BCG vaccination is performed at birth. T cell-based interferon-gamma (IFNγ) release assays (IGRAs) offer some advantages [8], [9], [10]. The most deterministic factors of possible *Mycobacterium tuberculosis* (Mtb) infection in children, include compatible clinical signs and symptoms, an X-ray indicative of TB and likelihood of infection due to known contact [11].

Mtb primarily resides in phagosomes within macrophages and hence its antigens are presented by class II major histocompatibility complex (MHC) molecules to CD4^+^ T cells. Studies in mice lacking CD4^+^ T cells have demonstrated the importance of this T cell subset in the control of TB [12], [13], [14]. CD4^+^ T cells activated by Mtb antigens may also become cytotoxic T lymphocytes (CTL) [15], [16], [17], [18], [19]. The critical role of CD4^+^ T cells in control of Mtb is further underlined by the high incidence of TB in HIV^+^ individuals whose CD4^+^ T cell compartment is affected [20]. CD4^+^ CTL express granzymes, FAS ligand (Fas-L), perforin and granulysin [21]. Granzymes and perforin directly kill target cells, whereas Fas-L induces apoptosis in target cells [18]. Granulysin expresses potent bactericidal activity and can directly attack Mtb in combination with granzymes and perforin [22], [23], [24]. Granulysin is expressed after 3–5 days following T cell activation [25]. It is generally present in human CTL, but a homologous molecule has not been described in mice [26]. We determined the expression of the cytotoxic granule protein granulysin in Mtb-specific CD4^+^ T cells after 7 days of *in vitro* stimulation. Thus, detection of granulysin-expressing CD4^+^ memory T cells could serve as basis for development of an immune marker for diagnosis of childhood and adolescent TB.

## Methods

### Ethics statement

The study using samples from children [EA2/0128/4] was approved by the Charité Ethics Committee University Hospital Berlin, Germany. T cells lines were generated from PBMC from adult TST^+^ donors and approved by the Charité Ethics Committee University Hospital Berlin, Germany [EA1/200/08]. Written informed consent was provided by all study participants or their legal guardians. Study participant groups are listed in Table 1.

**Table 1:** Characteristics of the study population.

Participants	LTBI	LTBI after therapy	Active TB patients	TB patients after therapy	NTM-infected children/adolescents	Healthy controls
**Total number**	**9**	**16**	**7**	**9**	**5**	**13**
Male	5	8	3	6	4	7
Female	4	8	4	3	1	6
Average range, years (median)	2–17 (13)	1–15 (10)	1–16 (14)	1–16 (6)	2–13 (7)	1–15 (9)

Tablenotes: Latent TB infection LTBI; nontuberculous mycobacteria NTM; tuberculosis TB.

### Study participants

To investigate the role of cytolytic T cells in childhood/adolescent TB, a total of 59 individuals (1–17 years; median age: 10 years) were recruited at the Department of Paediatric Pneumology and Immunology of the Charité University Hospital, Berlin, Germany. Individuals were characterized and defined as follows: Study participants with active TB had proven family contact to infectious TB and/or bacteriology (staining and/or culture and/or PCR), also positive TST and IGRA testing, as well as typical chest X-ray findings. Individuals with latent TB infection (LTBI) had proven family contact to patients with infectious TB and were also positive for TST and IGRA testing, but showed negative bacteriology and normal chest X-ray. Nontuberculous mycobacteria (NTM)-diseased participants showed cervical lymphadenitis, TST^+^ and negative IGRA. Bacterial culture for NTM after lymph node excision showed exclusive infection with *M. avium* and histological proof of epitheloid cell granulomatosis. In all cases, a TST was considered positive when induration was >5 mm. Quantiferon® TB-Gold In-Tube was used as IGRA according to manufacturer's instructions. An IGRA result was considered positive, when reaction to TB antigens minus the nil tube control was ≥0.35 IU/ml. Individuals with bacteriologically proven TB under antimycobacterial therapy were initially treated with a combination of isoniazid (INH), rifampicin (RMP) and pyrazinamide (PZA) [27]. For participants with advanced forms of TB, ethambutol (EMB) was included in initial treatment [28], [29]. All specimens showed full drug sensitivity. After termination of therapy, individuals underwent X-ray re-examination and bacteriological sputum negativity was confirmed. In the case of LTBI, chemoprophylaxis with INH was given for 9 months [30]. Controls were non-BCG-vaccinated individuals admitted to the hospital for non-TB-related diseases.

### Restimulation assay

The 7-day restimulation assay has been described previously [31]. In brief, peripheral blood mononuclear cells (PBMC) were isolated by density centrifugation (Biocoll, Biochrom) in accordance with manufacturer's instructions. PBMC (2×10^5^) were cultured in 200 µl RPMI 1640 medium (GIBCO, Invitrogen) containing 7.5% human serum (Sigma-Aldrich), 100 U/ml penicillin, 100 µg/ml streptomycin, 1 mM L-glutamine and 10 mM HEPES (all PAA laboratories) in 96-well round-bottom plates (NUNC). PBMC were stimulated with purified protein derivative (PPD) from Mtb (10 µg/ml) (Statens Serum Institute), recombinant Mtb fusion protein ESAT6-CFP10 (5 µg/ml) (courtesy of T.H. Ottenhoff/Leiden University) [32], recombinant EBNA1 protein from Epstein-Barr virus (EBV, 5 µg/ml), recombinant p65 protein from human cytomegalo-virus (HCMV, 5 µg/ml) or recombinant chimeric Chagas multiantigen from *Trypanosoma cruzi* (TcF, 5 µg/ml) (all Prospec). All stimuli were added at the beginning of culture and cells were kept at 37°C and 5% CO_2_. Cells were re-stimulated on day 6 with the same concentration of antigens, and Staphylococcal enterotoxin B from *Staphylococcus aureus* (SEB - Sigma-Aldrich) was also included as positive control for cytokine secretion (1 µg/ml). Brefeldin A (10 µg/ml) (Sigma-Aldrich) was added 4 h later and cells were cultured for an additional 12 h.

### Intracellular cytokine staining (ICS)

After the 7-day stimulation period, cells were fixed and permeabilized using BD cytofix/cytoperm™ (BD Biosciences) according to manufacturer's instructions. Cells were then washed twice in BD perm/wash™ solution (BD Biosciences), and incubated at 4°C for 45 min with fluorescence-conjugated antibodies directed against surface and intracellular proteins. The following antibodies were used: anti-CD4 APC-Cy7, anti-CD8 PerCP-Cy5.5, anti-CD45RO PE-Cy7, anti-CD45RA PE-Cy7, anti-CCR7 APC, anti-GranzymeB Alexa700, anti-HLA-DR APC, anti-CD107a/b FITC, anti-CD40L APC, anti-CTLA-4 APC (all BD Biosciences); anti-IFNγ Pacific Blue, anti-granulysin PE, anti-perforin FITC (all eBioscience); anti-CCR5 Alexa647 (AbD Serotec); anti-CXCR3 FITC (BioLegend). After staining, cells were washed twice in BD perm/wash™ solution and resuspended in PBS containing 5% FCS. Measurements were performed using the LSRII flow cytometer (BD Biosciences) and data was analyzed using FACS-Diva software 6.1 (both BD Biosciences) and FlowJo version 8.8.4 (Treestar).

### Proliferation assays

To measure the proliferation of T cells, PBMC were stained using CellTrace™ CFSE Cell Proliferation Kit (Invitrogen) according to manufacturer's instructions. Afterwards PBMC were plated and stimulated as described above.

### Generation of granulysin-expressing T cell lines

PBMC from adult TST^+^ donors were obtained and several wells were stimulated with PPD using the 7-day restimulation assay described above. After stimulation, cells were pooled and stained with CD3 APC, CD4 APC-Cy7, CD8 PerCp-Cy5.5, CD25 PE-Cy7, CXCR3 FITC (all BD Bioscience). Cells showing a CD3^+^, CD4^+^, CD8^−^, CD25^+^, CXCR3^+^ phenotype were sorted using a BD FACS ARIA 2 (Becton Dickinson). A limiting dilution of 0.3 and 0.5 cells per well in a 96-well plate (Nunc) was performed and cells were cocultured with 1×10^5^ autologous irradiated PBMC (28 grays). PPD (10 µg/ml) and recombinant IL-2 (40 U/ml) (Active Bioscience) were added in the first round of stimulation. Wells that showed visible growth were further expanded with irradiated PBMC, PHA (2.5 µg/ml) (Invitrogen) and IL-2 (40 U/ml).

### Assessment of anti-mycobacterial activity of T cell lines

Autologous PBMC were isolated as described above. Monocytes were then isolated using a Monocyte Isolation Kit II (Miltenyi) and 1×10^4^ monoyctes were plated per well in 96-well round-bottom plate in RPMI 1640 medium containing 7.5% human serum, 1 mM L-glutamine and 10 mM HEPES. Monocytes were infected with Mtb H37Rv at a multiplicity of infection (MOI) of 5 at 37°C and 5% CO_2_ for 24 h. After washing, different T cell lines were added at an effector∶target ratio of 10∶1. Plates were incubated for an additional 24 h at 37°C and 5% CO_2_. Afterwards cells were lysed with 100 µl of 0.1% Triton-X100 in PBS. Fifty µl of different dilutions of lysed cell suspension (1∶100; 1∶1,000; 1∶10,000; 1∶100,000) were plated on 7H11 agar plates. Plates were incubated at 37°C and colony forming units (CFU) were counted after 4 weeks.

### Data analysis

Statistical analyses were performed using GraphPad Prism© V.5a software. For analysis of the CD4^+^ CD45RO^+^ T cell compartment and granulysin expression, study groups were compared using the Mann-Whitney *U* test. The same test was used for the comparison of T cell lines lacking or expressing granulysin. Changes in median fluorescence intensity of the different immune markers, between granulysin-negative and granulysin-expressing CD4^+^ CD45RO^+^ T cells, and in memory phenotype of granulysin-expressing T cells, were compared using the Wilcoxon signed-rank test.

## Results

### Antigen-specific induction of granulysin-expressing CD4^+^ CD45RO^+^ T cells

**Figure 1:**
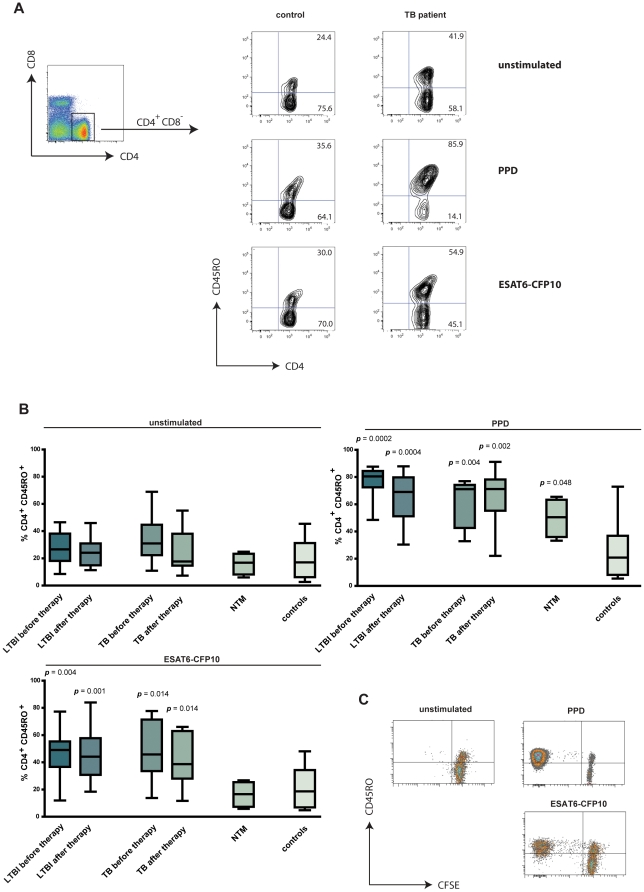
Increase of the CD4^+^ CD45RO^+^ memory T cell population after Mtb antigen stimulation. **A.** Representative density plot showing gating strategy for CD4 and CD8 lymphocytes (left). CD4^+^ T cells were further gated for CD45RO_low_ versus _high_ expression after: no stimulation (unstimulated), restimulation with purified protein derivative (PPD) or with ESAT6-CFP10. Representative examples for control (left) and active tuberculosis (TB) (right) are shown. **B.** Percentage of CD4^+^ CD45RO^+^ memory T cells unstimulated (left), after PPD (right) or ESAT6-CFP10 (bottom left) restimulation of 9 latent TB infection (LTBI) before therapy (light grey bar), 12 LTBI after drug therapy (dark grey bar), 7 active TB before drug therapy (light grey bar), 8 active TB after drug therapy (dark grey bar), 4 nontuberculous mycobacteria (NTM) (grey bar) and 13 controls without mycobacterial infection (white bar). Median represented by horizontal line, interquartile range by box, and range by whiskers. Significant difference of groups, in comparison to controls, is indicated (Mann-Whitney *U* test). **C.** Representative carboxyfluorescein diacetate succinimidyl ester (CFSE) proliferation dot plot of CD4^+^ T cells either unstimulated or after restimulation with PPD and ESAT6-CFP10, respectively.

**Figure 2:**
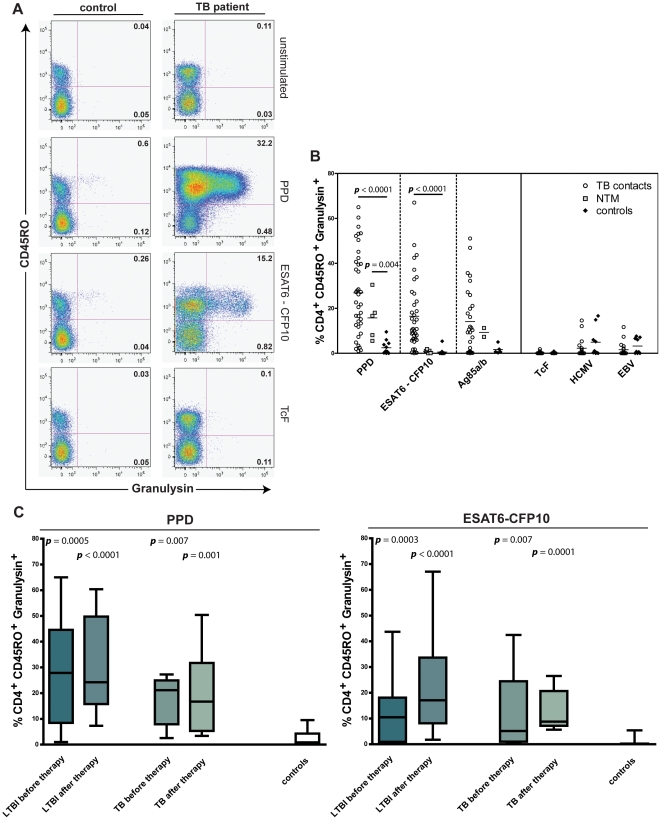
Frequency of CD4^+^ CD45RO^+^ granulysin^+^ memory T cells after Mtb antigen stimulation. **A.** Representative pseudo-color plot for CD45RO and granulysin expression by CD4^+^ T cells from control (left) and active tuberculosis (TB) (right) after 7-day restimulation. Peripheral blood mononuclear cells (PBMC) were unstimulated, stimulated with purified protein derivative (PPD), ESAT6-CFP10, or recombinant *Trypanosoma cruzi* antigen (TcF). **B.** Granulysin expression depicted for each antigen as percentage of CD4^+^ CD45RO^+^ memory T cells. Frequencies of 38 TB-experienced individuals (TB contacts) [latent TB infection (LTBI) and active TB before and after drug therapy] (circles), 5 nontuberculous mycobacteria (NTM) (squares), 12 controls without mycoabacterial infection (diamonds) are shown. PPD, ESAT6-CFP10, Ag85A/B, TcF, recombinant human cytomegalo-virus (HCMV) antigen Pp65, and recombinant Epstein-Barr virus (EBV) antigen EBNA-1, were used as antigens. **C.** Percentage of granulysin-expressing CD4^+^ CD45RO^+^ memory T cells from TB-experienced individuals: 10 LTBI before therapy (light grey bar), 12 LTBI after drug therapy (dark grey bar), 5 active TB (light grey bar), 9 active TB after drug therapy (dark grey bar) and 12 controls (white bar). Individual background values of unstimulated controls were subtracted. Median represented by horizontal line, interquartile range by box, and range by whiskers. Significant difference of groups, in comparison to controls, is indicated (Mann-Whitney *U* test).

TB-experienced children and adolescents, before or after treatment, presented higher frequency of CD4^+^ CD45RO^+^ T cells after 7-day restimulation with Mtb antigens when compared to controls (Figure 1A). The frequency of CD4^+^ CD45RO^+^ T cells in individuals infected with NTM and in controls was not changed after stimulation with the recombinant fusion protein ESAT6-CFP10 (Figure 1B). These two proteins are absent from many environmental mycobacteria as well as from BCG.. Proliferation experiments revealed expansion of CD4^+^ CD45RO^+^ T cells after stimulation with PPD or ESAT6-CFP10 (Figure 1C) and not with control antigens (data not shown). This indicates enlargement of the memory T cell pool rather than recruitment of naive T cells during stimulation. On the contrary, CD8^+^ CD45RO^+^ memory cells did not proliferate during 7-day restimulation and a significant reduction in CD8^+^ frequencies after antigen-specific stimulation was observed (data not shown). Next, we investigated the induction of CTL activation in the CD4^+^ CD45RO^+^ T cell compartment by determining granulysin expression. The gating strategy and expression of granulysin after antigen-specific stimulation of CD4^+^ T cells in a representative TB patient and control sample are displayed in Figure 2A. Participants with TB – regardless of drug therapy – were compared to those with NTM infection and to controls (Figure 2B). A significant induction of granulysin expression in antigen-specific memory T cells was detected for both TB patients (p<0.001) and NTM-infected individuals (p<0.01), but was absent in controls. Granulysin-expressing T cells were reduced after stimulation with ESAT6-CFP10 in Mtb-experienced individuals only (p<0.001). Stimulation with Ag85, a protein shared by Mtb, BCG and NTM, did not reveal significant differences between the three groups. Mtb-unrelated antigens (TcF, HCMV, EBV) were also included for further verification of antigen specificity. Stimulation with TcF did not result in granulysin expression. In some TB patients as well as in some controls, granulysin induction was observed after stimulation with EBV and HCMV antigen, however, at low frequency. Next, participants with Mtb infection were classified according to clinical status and anti-TB therapy (Figure 2C). Both individuals with active Mtb infection and LTBI showed a significant increase in granulysin-expressing memory T cells after PPD and ESAT6-CFP10 restimulation. A similar induction was detected in participants that underwent TB drug therapy.

Since CD4^+^CD45RO^+^ memory T cells contributed to the vast majority of granulysin-expressing cells after antigen restimulation (Figure 2A), we interrogated whether granulysin^+^ cells were derived from proliferating antigen-specific CD45RO^+^ memory T cells (Figure S1). Stimulation with PPD and ESAT6-CFP10 caused marked proliferation of CD45RO^+^ memory T cells, which were the main source of granulysin within the CD4^+^ T cell compartment. In contrast, the mitogen PHA induced strong T cell proliferation but almost no granulysin induction.

### Granulysin-expressing T cells reveal central and effector memory phenotype, are long-lived and coexpress IFNγ

**Figure 3:**
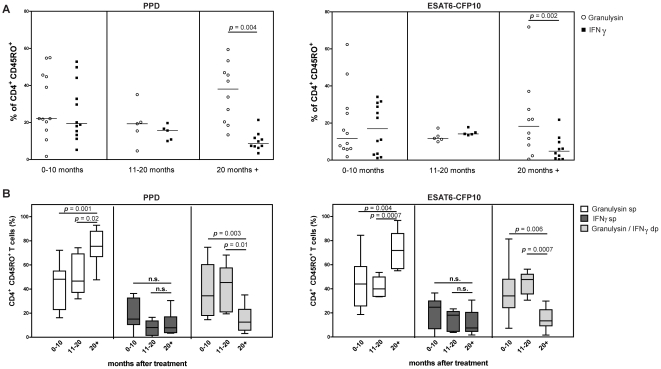
Antigen-specific induction of granulysin and/or IFNγ in CD45RO^+^ memory T cells of TB patients at different time points after drug therapy. **A.** Expression of granulysin (white circles) and interferon-gamma (IFNγ) (black squares) of CD4^+^ CD45RO^+^ memory T cells in peripheral blood mononuclear cells (PBMC) stimulated with purified protein derivative (PPD) (left) or recombinant ESAT6-CFP10 (right). Individual background values of unstimulated controls were subtracted. Medians for each group and statistical significance are indicated (Wilcoxon signed-rank test). **B.** Percentage of CD4^+^ CD45RO^+^ memory T cells expressing granulysin alone [granulysin single-positive (sp); white bars], IFNγ alone (IFNγ sp; grey bars) or both [granulysin/IFNγ double-positive (dp); light grey bars] after stimulation with PPD (left) or recombinant ESAT6-CFP10 (right). Median represented by horizontal line, interquartile range by box, and range by whiskers. Significance between different groups indicated (Mann-Whitney *U* test).

Further analysis revealed that after restimulation with ESAT6-CFP10, the majority of granulysin-expressing T cells presented an effector memory phenotype (CD45RA^−^ CCR7^−^) (p<0.01) (Figure S2). The same trend was observed after PPD restimulation, but results did not reach statistical significance. We next determined the longevity of this memory response after completion of TB drug therapy. To address this, we analyzed frequencies of antigen-induced granulysin- and IFNγ-expressing CD4^+^ CD45RO^+^ T cells in children recruited at different time points after TB drug therapy (Figure 3A). During the first 20 months after completion of drug therapy, both IFNγ and granulysin were observed. Children, who had completed TB drug therapy more than 20 months earlier, continued to show elevated frequencies of granulysin-expressing CD4^+^ CD45RO^+^ T cells, which were significantly higher compared to IFNγ (*p*<0.01). Hence, granulysin-expressing CD4^+^ T cells specific for Mtb are long-lived. The coexpression of granulysin and IFNγ was analyzed more precisely in the three different study groups (Figure 3B). Frequencies of IFNγ-producing single-positive memory T cells remained stable over time, whereas the proportion of IFNγ and granulysin double-positive memory T cells declined. However, the fraction of granulysin single-positive memory T cells increased markedly over time. These data suggest a shift over time from IFNγ-/granulysin double-positive memory T cells towards long-lived granulysin single-positive T cells after completion of drug therapy.

### Coexpression of surface markers and additional CTL effector molecules by granulysin-expressing CD4^+^ T cells

**Figure 4:**
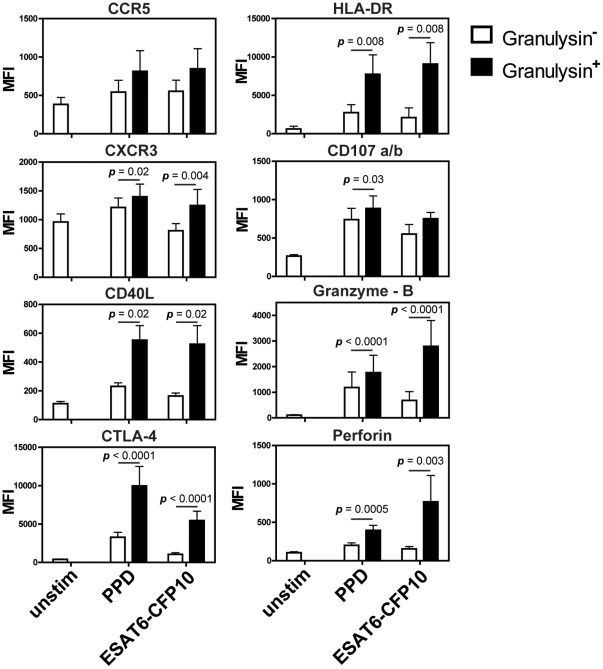
Median fluorescence intensity of different immune markers in granulysin-expressing CD4^+^ CD45RO^+^ memory T cells. PBMC (2×10^5^) from active TB and LTBI were stimulated with purified protein derivative (PPD), recombinant ESAT6-CFP10 or left unstimulated (unstim). Median fluorescence intensity (MFI) was measured in CD4^+^ CD45RO^+^ memory T cells and compared to T cells expressing granulysin upon restimulation (black bar) versus granulysin-negative T cells (white bar). Background expression is shown for unstimulated controls. Different numbers of patient samples were included in the analysis (CCR5 n = 9; CXCR3 n = 10; CD40L n = 7; CTLA-4 n = 15; HLA-DR n = 8; granzyme-B n = 21; perforin n = 12). Bars indicate arithmetic mean and overlying error bars represent standard error. Significant differences between granulysin^+^ and granulysin^−^ T cells are indicated (Wilcoxon signed-rank test).

Memory T cells are characterized by the expression of distinct sets of chemokine receptors. We analyzed expression of the chemokine receptors, CCR5 and CXCR3, characteristic for type 1 helper T cells [33], [34], as well as activation markers (HLA-DR, CTLA-4, CD40L) [35], [36], [37] and additional cytotoxic markers (granzyme B and perforin) in granulysin-expressing T cells upon antigen stimulation (Figure 4). CXCR3 was significantly elevated on granulysin^+^ T cells, whereas CCR5 expression remained stable. Antigen-specific CD4^+^ CD45RO^+^ T cells also surface-expressed activation markers CD40L, CTLA-4 and HLA-DR at elevated intensity. Moreover, the cytotoxic proteins granzyme-B and perforin were highly induced in granulysin-expressing CD4^+^ T cells (Figure 4).

### CD4^+^ T cell lines reduce Mtb burden in infected target cells in a granulysin-dependent fashion

**Figure 5:**
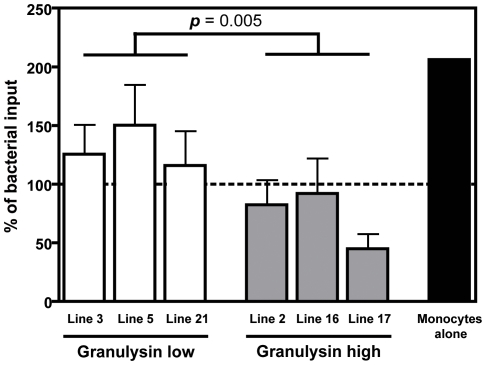
Differential effect of granulysin high- and low-expressing T cells on Mtb replication. Monocytes (1×10^4^) were infected with *Mycobacterium tuberculosis* (Mtb) 37Rv (multiplicity of infection, MOI = 5) for 24 h. Infected cells were incubated alone (black bar), or with autologous T cells expressing high and low levels of granulysin (effector∶target ratio = 10∶1). Three granulysin-low (white bars) and three granulysin-high (grey bars) T cell lines were used. After 24 h of co-culture, cells were lysed and lysates were serially diluted and plated into 7H11 agar. Colony forming units (CFU) were counted after 4 weeks. Percentages of bacterial input were calculated and are arranged in granulysin-high and -low groups. Groups were compared by Mann-Whitney *U* test. Dotted line represents bacterial inoculum (5×10^4^ bacteria, 100%).

Granulysin has been demonstrated to reduce Mtb burden in infected target cells [22]. We therefore infected monocytes and cocultured them with autologous granulysin high or low T cell lines (Figure 5). Growth of Mtb was significantly decreased in cultures with granulysin-high, compared to granulysin-low, T cell lines reemphasizing their important role in the control of Mtb replication.

## Discussion

CTL play an essential role in the immune control of TB [18]. Principally, CTL use two major pathways for elimination of infected target cells. Surface expression of FAS-L causes apoptosis in target cells expressing FAS. Exocytosis of granular content into the immunologic synapse, between CTL and target cells, causes lysis [38]. Effector proteins contained within cytotoxic granules include perforin, granzymes and granulysin [39]. Granulysin has direct antimicrobial activity against a wide spectrum of bacteria, parasites and fungi [22]. Notably, even though CTL activity is typically associated with CD8^+^ T cells, several reports have demonstrated Mtb-associated CTL activity in CD4^+^ T cells in TB [21], [40], [41]. Here we analyzed granulysin-expressing CD4^+^ T cells in childhood/adolescent TB and harnessed this T cell set for differential diagnosis of TB [31]. We detected profound induction of memory T cells defined as CD4^+^ CD45RO^+^ T cells after restimulation with Mtb antigens (Figure 1A,B). Restimulation significantly increased the frequencies of CD4^+^ CD45RO^+^ memory T cells in all study groups with Mtb infection, with or without TB drug therapy.

Study participants infected with NTM showed heightened frequencies of memory T cells after restimulation with the crossreactive PPD, but not after restimulation with Mtb-specific ESAT6-CFP10. In controls, frequencies of memory T cells remained unchanged after restimulation.

A possible reason for the marked induction of CD45RO^+^ T cells during 7-day culture could be the recruitment of naive cells to the memory compartment. We consider this possibility unlikely because CD45RO^+^ memory T cells were not induced by the Mtb-specific antigen ESAT-CFP10 in controls and in individuals infected with NTM. To verify whether only T cells of the memory compartment were induced by restimulation, we performed proliferation assays, which provided compelling evidence that memory T cells exclusively proliferated after restimulation with PPD and ESAT6-CFP10 (Figure 1C). Induction of CD8^+^ CD45RO^+^ memory cells could not be detected and frequencies of CD8+ T cells decreased significantly after stimulation (data not shown). Granulysin expression by T cells plays a crucial role in immune defence against TB [18], [42]. Yet, the 7-day restimulation assay is highly sophisticated and hence, in its present form, not ready for point-of-care diagnosis of childhood/adolescent TB.

Granulysin induction in memory T cells of study participants infected with Mtb, with or without TB drug therapy, was profound (*p*<0.0001) after PPD or ESAT6-CFP10 restimulation as compared to controls (Figure 2B). This elevated granulysin remained high in individuals newly diagnosed with active TB (*p*<0.001) or LTBI (*p*<0.01) as compared to controls (Figure 2C). Better measures for diagnosis of childhood TB are urgently needed [11]. Our data favour a 7-day *in vitro* restimulation assay for TB diagnosis in children. Flow cytometry has been exploited for TB diagnosis by numerous groups [43], [44], [45]. Most studies focused on intracellular IFNγ and tumour necrosis factor-alpha (TNFα) expression after restimulation, which increased sensitivity over IGRA. Despite the fact that our study was performed in a low-incidence high-income country, where the number of study participants was limited, our findings, suggest the inclusion of granulysin in flow cytometry-based assays as a diagnostic marker for childhood TB. We did not detect any correlation between antigen-specific granulysin expression of CD4+ CD45RO+ memory T cells and age distribution. Since age has been reported as one of the most important risk factors in childhood/adolescent TB, further studies comparing granulysin in different age groups would be crucial [46].

Consistent with previous studies from our group, the 7-day *in vitro* restimulation assay caused profound reactivation of CD45RO^+^ memory T cells [31]. These CD4^+^ CD45RO^+^ memory T cells were the main source of granulysin as indicated by exclusive expression in CD4^+^ CD45RO^+^ T cells undergoing cell division (Figure S1).

Additional experiments revealed an equal contribution of central and effector memory T cells after PPD re-stimulation and a preponderance of effector memory CD4^+^ T cells after ESAT6-CFP10 restimulation (Figure S2). This could be explained by superior processing of the recombinant protein resulting in faster activation and transition of resting central effector memory T cells into an effector memory phenotype. These findings are consistent with the data of others [47].

Di Liberto et al. [48] suggested that serum concentration of granulysin in children could be exploited for monitoring outcome of drug therapy in TB. In this study the serum concentration of granulysin before and after drug therapy differed. This effect was not seen at the T cell level in our study (Figure 2). On the contrary, our data revealed a constant high frequency of granulysin^+^ memory T cells even 20 months after termination of drug therapy (Figure 3A). Our data emphasize that granulysin is expressed by long-lived memory T cells. Hence its potential as immunologic marker for long-lived memory T cells in TB, should be exploited. In support of this notion, a recent report has shown that CD4^+^ and CD8^+^ T cells of BCG-vaccinated infants express granulysin after *ex vivo* stimulation with either BCG or PPD [49]. Moreover, vaccination with recombinant *M. smegmatis*, coexpressing granulysin and IL-12, reduced pulmonary CFU in lungs of Mtb-challenged mice [50].

A proportion of the memory T cell pool coexpressed IFNγ and granulysin (Figure 3B). Coexpression of these two key molecules in immune defense against Mtb has previously been shown for T cell clones [41], [51]. Our study identifies, for the first time, this T cell subset in children/adolescents after TB drug therapy and a shift from granulysin/IFNγ double-positive CD4^+^ T cells towards single-granulysin^+^ memory CD4^+^ T cells, over time (Figure 3B).

The phenotype of human CD4^+^ CTL has not been investigated in detail. Thus, we characterized additional surface markers and other cytotoxic proteins in granulysin^+^ CD4^+^ T cells (Figure 4). Amongst chemokine receptors characteristic for type 1 helper T cells, CXCR3 was significantly upregulated on granulysin^+^ T cells, whereas CCR5 expression remained unchanged. Bastian et al. [47], found elevated expression of CXCR3 as well as CCR5 on the surface of CD4^+^ CTL after stimulation with crude Mtb cell wall extracts. Increased expression of activation markers, including CD40L, CTLA-4 and HLA-DR, indicates that granulysin-producing CD4^+^ T cells are highly activated (Figure 4). Expression of the costimulatory surface molecule CD40L on CD4^+^ CTL has not been described before. Expression of CTLA-4 on Mtb-specific CD4^+^ T cell clones has been found to inhibit lytic activity after crosslinking [52]. Thus, CTLA-4 is likely involved in the regulation of CD4^+^ CTL.

Our experiments focused on granulysin expression by CD4^+^ CTL. Two other major cytolytic proteins in the lytic granule, perforin and granzyme B, were found to be coexpressed with granulysin (Figure 4) [22], [39]. This confirms previous observations showing that CD4^+^ T cells upregulate perforin and granzyme B after *in vitro* restimulation with Mtb [21]. This induction of granzyme B and perforin supports a cytotoxic function of these granulysin-expressing memory CD4^+^ T cells in TB. This notion could be further strengthened by the co-culture experiments with granulysin-expressing CD4^+^ T cell lines (Figure 5). As discussed above, the second pathway for target cell killing by CTL is mediated by CD95/CD95L interactions [53]. This route of induced apoptotic cell death can be excluded in our assay since CD95L on the cell surface of granulysin^+^ T cells was not detected (data not shown). Consistent with previous studies we assume that CD4^+^ CTL primarily use the cytotoxic granule mechanism rather than the CD95-dependent pathway for target cell killing [54], [55], [56].

In conclusion, we describe antigen-specific expression of granulysin by long-lived central and effector memory CD4^+^ T cells in Mtb-infected children and adolescents. Hence, we suggest further exploring the potential of this immune marker for diagnosis of childhood/adolescent TB.

## Supporting Information

Figure S1 **Induction of granulysin exclusively in proliferating antigen-specific CD45RO^+^ memory T cells.** Representative dot plots (top) and histograms (bottom) of frequencies of carboxyfluorescein diacetate succinimidyl ester (CFSE)_high_ and CFSE_low_ populations and percentages of CD4^+^ CD45RO^+^ T cells expressing granulysin in active tuberculosis (TB) after restimulation with purified protein derivative (PPD) or ESAT6-CFP10. As positive control (far right) cells were incubated with 5 µg/ml phytohemagglutinin (PHA). Results showing are representative for six independent experiments.
(TIF)

Figure S2 **Memory phenotype of granulysin-expressing CD4^+^ T cells in children/adolescents.** Peripheral blood mononuclear cells (PBMC) of active tuberculosis (TB) and latent TB infection (LTBI) were restimulated with purified protein derivative (PPD) (left) or ESAT6-CFP10 (right). Cells were stained for granulysin, CD45RA and CCR7 and granulysin^+^ cells grouped based on distribution of surface markers. Means for each group and significant differences between central memory T cells (T_cm_) and effector memory T cells (T_em_) are indicated (Wilcoxon signed-rank test).
(TIF)
