# Supplementary figures and images for: Granulysin-Expressing CD4+ T Cells as Candidate Immune Marker for Tuberculosis during Childhood and Adolescence
Source: PLoS One. 2011 Dec 27;6(12):e29367. doi: 10.1371/journal.pone.0029367 (PMC3246496; doi:10.1371/journal.pone.0029367)

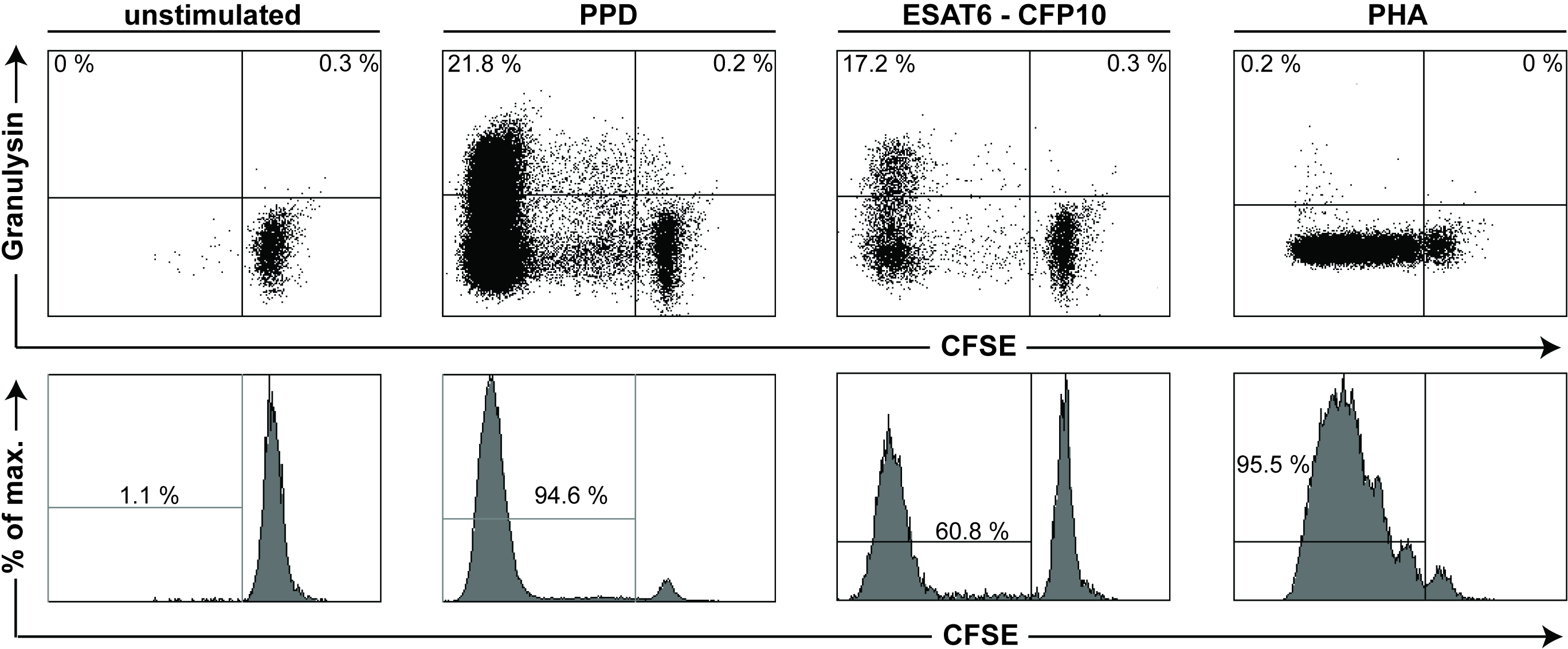

Supplement: Figure S1 — Induction of granulysin exclusively in proliferating antigen-specific CD45RO+ memory T cells. Representative dot plots (top) and histograms (bottom) of frequencies of carboxyfluorescein diacetate succinimidyl ester (CFSE)high and CFSElow populations and percentages of CD4+ CD45RO+ T cells expressing granulysin in active tuberculosis (TB) after restimulation with purified protein derivative (PPD) or ESAT6-CFP10. As positive control (far right) cells were incubated with 5 µg/ml phytohemagglutinin (PHA). Results showing are representative for six independent experiments. (TIF) [file pone.0029367.s001.tif]

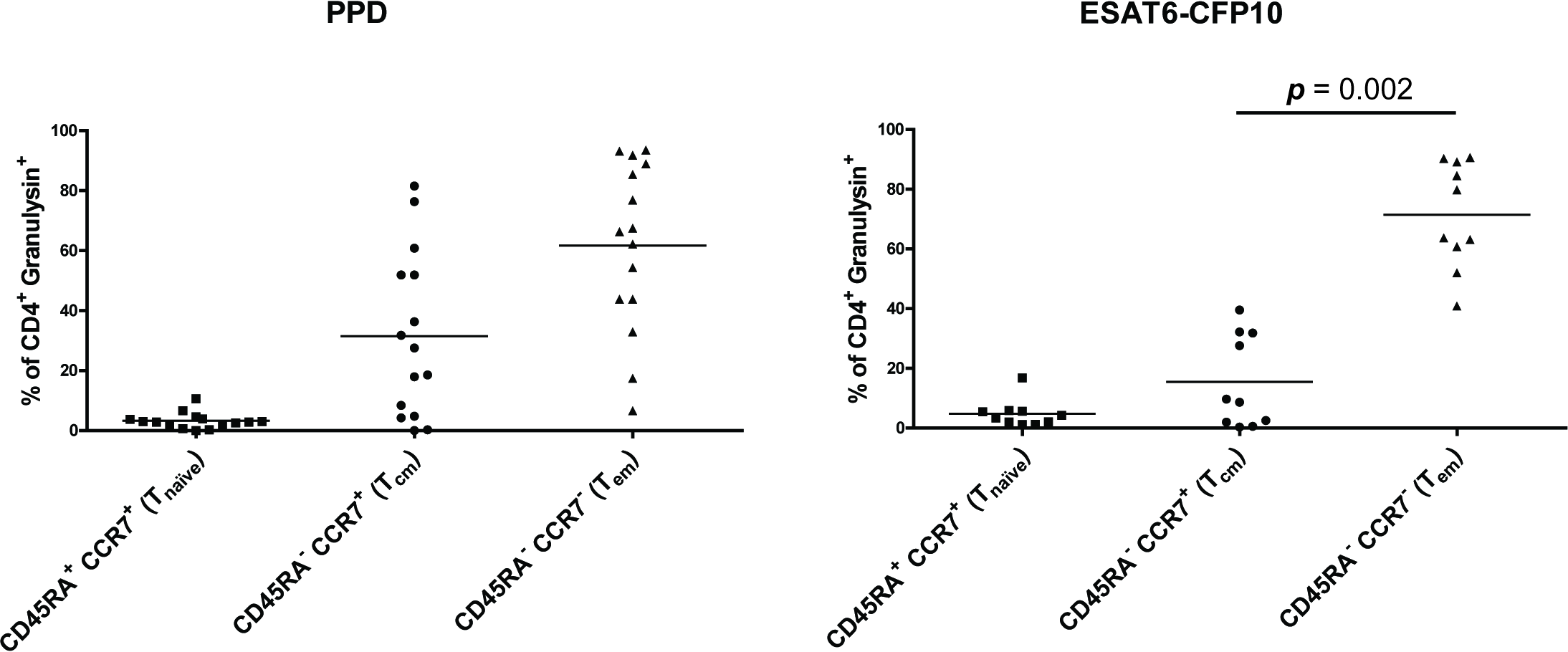

Supplement: Figure S2 — Memory phenotype of granulysin-expressing CD4+ T cells in children/adolescents. Peripheral blood mononuclear cells (PBMC) of active tuberculosis (TB) and latent TB infection (LTBI) were restimulated with purified protein derivative (PPD) (left) or ESAT6-CFP10 (right). Cells were stained for granulysin, CD45RA and CCR7 and granulysin+ cells grouped based on distribution of surface markers. Means for each group and significant differences between central memory T cells (Tcm) and effector memory T cells (Tem) are indicated (Wilcoxon signed-rank test). (TIF) [file pone.0029367.s002.tif]
